# Therapeutics

**Published:** 1878-09

**Authors:** 


### Therapeutics.

An Additional Development of External Metallotherapy.—Dr. R. Vigouroux.—(*Le Progrès Médical.*)

A reference has been made in the Journal to the work being done by Drs. Charcot, Vigouroux and Burg, by the application of metallic and other discs to different places in the human body in an abnormal condition arising from the vaguely defined affection known as hysteria. The result of this work is the establishment of the fact that certain hyperæsthesic and anæsthesic areas become greatly modified on the application of these discs—the proper disc, either of gold, silver, copper, zinc, iron, etc., being ascertained by actual experiment. The modification, in some cases, having resulted in the cure of some hysterical patients who had suffered for years without relief. The difficulty that has hitherto been experienced, has been the fugitiveness of the relief. Dr. Vigouroux has discovered that by seizing the moment when the relief is experienced, and placing upon the already applied disc another disc, from the application of which the patient previously received no benefit, the effect becomes if not permanent, greatly prolonged while the two are applied. Moreover, the same effect follows when the second or neuter disc is applied merely in the immediate neighborhood of the active disc. Before we cite the two cases presented by Dr. V., it is necessary to state that the same effect follows when the modification of the abnormal condition is obtained by galvanic or statical electricity.

Examples: A patient affected with hemianæsthesia of the right side, is placed on an insulated stool. Under the influence of a simple electric bath the hemianæsthesia and achromatopsia (color-blindness) passes to the left side, then disappears. At that moment Dr. V. fixes upon the right arm of the patient (sensible to tin) a disc of brass about the size of a button. The next day the anæsthesia and color-blindness return, but on the left side.

With another patient affected with total anæsthesia, the sensitiveness being restored with the same means, usually remains only a few minutes after the operation, but a plate of brass—the patient is sensitive to zinc—being placed upon both arms, the sensitiveness remains the whole day, and the patient is advised of the return of the anæsthesia by a special sensation of fluttering (*une sensation d' abattement.*)

Another patient, with the application of a little disc of gold-plated copper—she was sensitive to gold—retained her sensitiveness for eight days, but the anæsthesia returned as soon as the disc was removed. A similar experiment with the same result was repeated sometime afterwards.

Dr. V. promises to furnish the details shortly. We shall look forward to the fulfillment of the promise with great interest.

---

According to *Harper's Weekly*, the members of the New York Medical Club were invited to an entertainment, a few years ago, by Dr. H. D. Paine, of that city, in the following terms:

"SCIENS, SOCIALITE, SOBRIETE."

Doctores,—Ducum nex mundi nitu Panes; tritucum at ait. Expecto meta fumen tu te & eta beta pi. Super attento, uno. Dux, hamor clam pati, sum parates, homine, ices, jam, etc. Sideror hoc. Anser.

"FESTO REASONAN FLOAS SOLE."

